# HTLV-1 ORF-I Encoded Proteins and the Regulation of Host Immune Response: Viral Induced Dysregulation of Intracellular Signaling

**DOI:** 10.1155/2015/498054

**Published:** 2015-10-18

**Authors:** Carolina Rosadas, Marzia Puccioni-Sohler

**Affiliations:** ^1^Universidade Federal do Rio de Janeiro (UFRJ), Rua Rodolpho Paulo Rocco 255, 3° Andar, Cidade Universitária, Ilha do Fundão, 21941-913 Rio de Janeiro, RJ, Brazil; ^2^Universidade Federal do Estado do Rio de Janeiro (UNIRIO), Rua Mariz e Barros 775, Tijuca, 20270-901 Rio de Janeiro, RJ, Brazil

## Abstract

The human T-cell lymphotropic virus type 1 (HTLV-1) is a retrovirus associated with both proliferative and inflammatory disorders. This virus causes a persistent infection, mainly in CD4+ T lymphocyte. The ability to persist in the host is associated with the virus capacity to evade the immune response and to induce infected T-cell proliferation, once the HTLV-1 maintains the infection mainly by clonal expansion of infected cells. There are several evidences that ORF-I encoded proteins, such as p12 and p8, play an important role in this context. The present study will review the molecular mechanisms that HTLV-1 ORF-I encoded proteins have to induce dysregulation of intracellular signaling, in order to escape from immune response and to increase the infected T-cell proliferation rate. The work will also address the impact of ORF-I mutations on the human host and perspectives in this study field.

## 1. Introduction

Human T-cell lymphotropic virus type 1 (HTLV-1) was first isolated in the early 80s, in a patient with a T-cell lymphoma [1]. Nowadays, it is known that HTLV-1 persistently infects CD4+ T cells throughout the individual's whole life. It is estimated that HTLV-1 currently infects approximately 5–10 million individuals throughout the world [2]. HTLV-1 infection is usually asymptomatic; however, it can cause mainly an aggressive and fatal CD4+ T-cell malignancy, the adult T-cell leukemia (ATL), and a neurodegenerative and disabling disorder of central nervous system, HTLV-1 associated myelopathy/tropical spastic paraparesis (HAM/TSP) in up to 5% of infected individuals. Therefore, this virus is associated with both proliferative and inflammatory disorders in humans [2–5]. The mechanisms that lead to clinical manifestation in some individuals are still poorly understood. In this context, it is known that high proviral load is associated with disease outcome [6–10].

The viral genome is approximately 9 kb long and contains the typical retroviral structural and enzymatic genes* gag*,* pol*, and* env*. In addition, the provirus presents a unique* pX* region, which contains the regulatory and nonstructural genes. Thus, the 3′ end of the viral genome expresses alternatively spliced mRNAs encoding proteins from open reading frames (ORFs) I–IV. ORF-I encodes the p12^I^ protein. This protein is highly hydrophobic and presents four SH3 binding motifs, one leucine zipper domain, one leucine zipper-like domain, and two putative transmembrane domains (TM1 and TM2) [11, 12]. The viral p12^I^ protein undergoes complex posttranslational modifications. It undergoes proteolytic cleavage at two different sites: at position 9 (L9↓S10) and at position 29 (G29↓L30). These yields two protein forms: p8 and p12 (8-kDa and 12-kDa forms) which presents different cellular localization and function, while 12-kDa (p12) remains in the endoplasmatic reticulum (ER) and* cis*-Golgi compartment the 8-kDA form (p8) traffic to cell surface. Indeed, early studies have shown that the p12 cDNA expression yields two proteins [13]. Currently, we know that these two proteins are p8 and p12.

**Figure 1:**
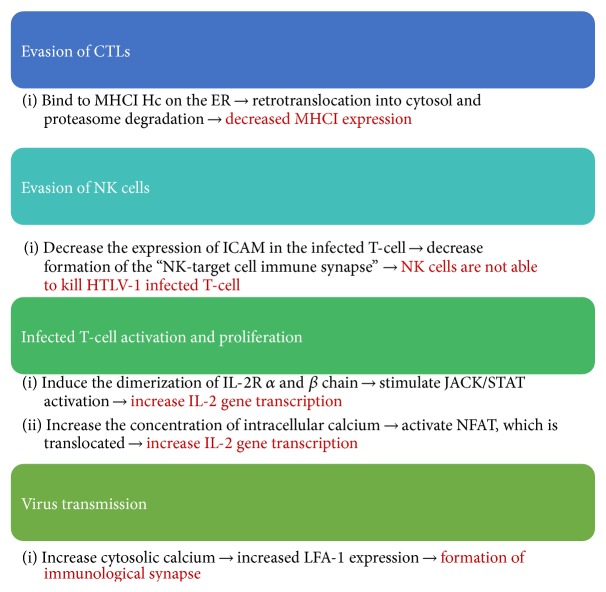
Summary of the host cell alterations induced by HTLV-1 ORF-I encoded proteins and their consequences.

The ability of HTLV-1 to achieve a persistent infection may be associated with the virus capacity to evade the immune response. The immune evasion may be also associated with a high viral replication rate that can lead to a high proviral load, which, in turn, is related with disease manifestation [6–9]. A previous analysis demonstrated that higher HTLV-I proviral DNA load in cerebrospinal fluid (CSF) was associated with absence of intrathecal synthesis of HTLV-I antibodies [10, 14]. In this context, some studies demonstrated that ORF-I encoded proteins are associated with the ability of immune evasion of HTLV-1 infected cells as also with infected T-cell proliferation. The present study will address the molecular mechanism of p12 and p8 HTLV-1 protein to regulate the host immune response (Figure 1), indicating the current status of the knowledge in this field, pointing new possibilities to achieve a better understanding of the role of HTLV-1 ORF-I proteins to infected individuals. The majority of the studies related to cellular alterations, caused by the HTLV-1 p12, were based on ORF-I silencing or depletion. Currently we know that both p12 and p8 are affected by this technique. Therefore, in the present paper, we will address both proteins as ORF-I encoded proteins. Previous studies that mentioned p12 protein may also be referring to p8 functions.

## 2. HTLV-1 ORF-I Encoded Proteins and the Modulation of Cytotoxic T Lymphocytes (CTLs) Response

The recognition of an infected cell by CTLs is made through the specific interaction between the T-cell receptor (TCR) and the MHC-I molecule presenting an antigen.

The peptides (antigens) are generated by the proteasome in the cytoplasm. After, they are transported by TAP (transporter associated with antigen processing) to the ER, where they are conjugated to MHC-I molecule. These MHC-I-antigen complexes are transported to the infected cell surface for CTL recognition and activation. MHC-I is assembled in the ER lumen and presents a heavy chain (Hc) and *β*2-microglobulin. Several pathogens developed many strategies targeting MHCI molecule in order to escape from CTLs response [15]. It was shown that HTLV-1 ORF-I proteins can bind to MHC-I Hc in the ER, causing the retrotranslocation to the cytoplasm followed by proteasome degradation. This results in a decreased expression of MHC-I at the cell plasma membrane and consequently evasion from CTLs [15].

The down modulation of MHC-I expression, on the other hand, makes HTLV-1 infected cells susceptible to natural killer (NK) response. However, this virus also presents mechanisms to evade NK cell activity. This ability can be also mediated by ORF-I encoded proteins and will be discussed in the next topic.

In contrast, p12 expression did not alter the expression level of MHC-I and MHC-II in infected immortalized T cells. This data indicates that ORF-I encoded proteins may modulate the MHC expression only during the early stage of infection [16].

## 3. HTLV-1 ORF-I Encoded Proteins and the Modulation of NK Cell Response

The NK cell activation is the result of the integrated balance between the stimulation of activating and inhibitory NK receptors. Therefore, a prior recognition of the pathogen is not necessary for the NK cell activation by the infected cell. NK cells are activated by activating ligands present on the cell surface. As uninfected cells can also express these ligands, the noninfected cells have to be able to dampen NK cell cytotoxicity. This is achieved by the engagement of specific molecules, such as MHCI, to the inhibitory receptors (iNKRs) [17–19]. Therefore, if the virus induces a down modulation of MHCI in the target cell, it can be identified and further destroyed by NK. In this context, the viral Tax protein can cause an overexpression of MHCI molecules to avoid NK cell response [20]. Therefore, it seems that p12 and Tax proteins present contradictory roles in the intracellular signaling pathways, once it was demonstrated that HTLV-1 ORF-I encoded proteins cause a down modulation of MHC-I expression.

Another important point to NK cell activity is the formation of the “NK-target cell immune synapse.” This strong adhesion between NK and the target cell is mediated by integrins present in NK and their natural ligands available in the target cell. This is critical to trigger NK cell response. The leukocyte function antigen 1 (LFA-1) is one of those integrins that can be found in NK cell surface and binds to intercellular adhesion molecules (ICAMs). The ICAMs are expressed by several cells, including leukocytes. Besides the formation of immune synapses, LFA-1 can also trigger intracellular signaling for cytotoxic granule polarization, which play an important role in directing the machinery of NK to target cell. An* in vitro* study, using a lentivirus vector encoding HTLV-1 p12, demonstrated that NK cells are not able to kill HTLV-1 infected primary CD4+ T cells. This inability was associated with the down modulation of ICAM-1 and ICAM-2 in the infected cell, which results in a decreased ability to form the immune synapse. This alteration was induced by HTLV-1 p12. In the same study, neither NCR nor NKG2D ligands were expressed by HTLV-1 infected T cells [19]. The exact mechanism, however, is not known.

## 4. HTLV-1 ORF-I Encoded Proteins and Infected T-Cell Activation and Proliferation

It is know that HTLV-1 infection is maintained mainly by the clonal expansion of infected T-cell [4]. In this context, HTLV-1 ORF-I encoded proteins may stimulate T-cell proliferation by the activation of the Jack/STAT pathway. Therefore, these proteins decrease the amount of IL-2 requirement for T-cell proliferation increasing the HTLV-1 infected cell proliferation [12, 21, 22]. Another mechanism of the fact that p12 has to induce T-cell proliferation involves the activation of the transcription factor NFAT, by increasing the cytoplasmic Ca^2^. An increase in the concentration of intracellular calcium activates the phosphatase calcineurin that dephosphorylates NFAT, which is translocated to nucleus and induces IL-2 expression [23]. Both NFAT and STAT5 can bind to IL-2 promoter, increasing the expression of this important protein to HTLV-1 infection [12, 21, 24, 25].

In fact, studies with indirect immunofluorescence and electron microscopy showed that p12 is retained in ER. The same study showed that p12 is associated with calreticulin and calnexin, which are resident ER proteins and regulate the calcium storage [26]. This binding property of p12 to those proteins modulates NFAT activation [27]. In addition, using a lentiviral vector that expressed p12 in Jurkat cells, it was demonstrated that ORF-I encoded protein enhances IL-2 production in a calcium pathway-dependent manner and increases NFAT-mediated reporter genes activities during T-cell receptor (TCR) stimulation. However, in PBMCs, p12 increased IL-2 production only with the concomitant stimulation of phorbol ester and not during TCR stimulation. This difference may be associated with different responses of the transformed Jurkat cells and primary PBMCs. The same study showed that p12 truncated mutants showed different patterns of NFAT activation [28]. In contrast, ORF-I encoded proteins (not known if p12, p8, or both) can also bind to calcineurin [29], leading to inhibition of NFAT through competitive-binding. Thus, ORF-I encoded proteins can regulate NFAT in a positive or negative manner [23].

Moreover, the p12 protein binds to the cytoplasmic domain of the IL-2 receptor (IL-2R) in the ER [30]. The p12 binding to the cytoplasmic domain of IL-2R occurs in a region involved in transduction of IL-2 signal. This causes an increase in signal transducer and activator of transcription 5 (STAT5) DNA binding activity even in the absence of IL-2. Besides, there is an increase of STAT5 phosphorylation in primary PBMCs and decreases in the IL-2 requirement for T-cell proliferation [31]. In support of this finding, at low IL-2 concentrations, ORF-I encoded proteins showed to increase the proliferation of HTLV-1 infected cells [32]. In the absence of exogenous mitogens, ACH.p12 was less efficient to infect quiescent PBMCs [33].

All these data together clearly demonstrate that ORF-I encoded proteins induce intracellular signaling modification that result in a decreased IL-2 requirement for T-cell proliferation. These HTLV-1 accessory proteins seem to confer a growth advantage to infected T cells, mainly during the early stage of infection, before the immortalized T-cell state [27].

## 5. HTLV-1 ORF-I Encoded Proteins and Virus Transmission

HTLV-1 maintains the infection almost exclusively by the clonal expansion of infected T-cell [34]. Therefore, the stimulation of T-cell proliferation that can be modulated by ORF-I encoded proteins, as mentioned before, contributes to virus persistence. However, in a recent infection of a new host, HTLV-1 has to produce new virions and actively infect new host cells [34–36]. HTLV-1 can be transmitted through cell-to-cell junctions, also known as virological synapses [37] and/or by virofilm structures [38]. The virological synapses mimic immunological synapses. Regarding this issue, it is known that LFA-1 expression is associated with T-cell activation, mediates T-cell adhesion and lymphocyte migration into tissues, and participates in immunological synapse. The activation of LFA-1 can be calcium-mediated [39]. Studies showed that p12 can induce calcium release from ER, increasing the calcium concentration in cytosol thus activating T-cell [25]. In addition, T cells infected with the mutant ACH.p12^I^ clone that is not able to produce p12, although can express LFA-1, exhibited less LFA-1 mediated adhesion than T cells infected with wild type clone (ACH). In the same study the authors also demonstrated that the expression of p12 in Jurkat T cells enhanced the cell adhesion mediated by LFA-1 in a calcium dependent manner, once it was inhibited with the addition of calcium chelator, calcium channel blocker, and an inhibitor of the calcium dependent protease calpain [40]. Reinforcing this finding, although p12^+^ and p12^−^ cell lines produced similar amounts of HTLV-1 particles in the supernatant of culture cells, ORF-I encoded proteins expression enhanced virus transmission more than twofold when compared to infected cells lacking p12. The same study showed that p12 expression increased the rate of syncytium formation [32]. More recent studies demonstrated that this increased clustering of LFA-1 is actually mediated by p8 and not by p12 [23, 41]. Therefore, ORF-I encoded proteins seem to facilitate HTLV-1 transmission through virological synapses. This spread mechanism is also important to prevent the immune response against HTLV-1. Furthermore, ORF-I encoded proteins could also indirectly increase HTLV-1 production, once IL-2 increases CREB/ATF and Tax transcription [42].

## 6. The Impact of HTLV-1 ORF-I Encoded Proteins on* “In Vitro”* HTLV-1 Infection and in Animal Model

Several studies addressed the role of HTLV-1 ORF-I proteins to viral infection using* in vitro* studies and animal model. A study conducted by Collins and colleagues using a HTLV-1 molecular clone that was not able to produce p12 (ACH.p12^I^) demonstrated that this virus was infectious* in vitro*; however, it was not able to establish a persistent infection in rabbits. Indeed, after the ACH.p12^I^ inoculation, the animals presented a reduced anti-HTLV-1 humoral response compared to those infected with the full-length clone ACH. Moreover, p19 antigen was not detected in peripheral blood mononuclear cells (PBMCs) of ACH.p12^I^ infected animals and there was only a transient detection of HTLV-1 proviral DNA in PBMCs in those animals [43]. It is important to highlight that the differences observed between the* in vitro* and* in vivo* studies may be associated with the stimulation of cell cultures with IL-2 and phytohemagglutinin. Other studies also demonstrated that different molecular clones lacking ORF-I did not alter the HTLV-1 capacity to infect, replicate, and immortalize T-cells in cell cultures [44, 45]. In contrast, studies showed that in limited IL-2 environments, ORF-I deletion caused a decreased virus transmission in PBMCs [46]. Indeed, HTLV-1 ORF-I encoded proteins seem to influence IL-2 production and response in infected T cells. Regarding the role of ORF-I encoded proteins in animal model, a recent work demonstrated that both p12 and p8 are essential for persistence and spread. HTLV-1 molecular clone that expresses only p12 and was not able to express p8 was not infectious to nonhuman primates [47].

## 7. The Impact of ORF-I Mutations on Viral Replication and Human Host

ORF-I encoded proteins are expressed in naturally infected individuals. This was evidenced by the detection of ORF-I mRNA in HTLV-1 infected cells from HAM/TSP and ATLL patients and from asymptomatic carriers [48, 49]. In addition, recombinant p12 is recognized by serum antibodies from HTLV-1 infected individuals [50]. These HTLV-1 proteins are clearly associated with immune evasion and T-cell proliferation. Consequently, a mutation in this region may influence the virus capacity to escape from immunological response and may induce or inhibit the infected T-cell proliferation. At first, a lysine to arginine change at position 88 of p12 protein found in natural alleles was associated with HAM/TSP development. This amino acid substitution affects the stability of the protein, with the p12R allele being more stable than the p12K. First, p12K88 was described just in HAM/TSP samples [51]. However, other studies demonstrated that this mutation was associated with geographic distribution rather than disease progression [52–54].

G29S represents another known mutation. It inhibits the production of p8 protein at the cleavage site position of p12. All these data together highlighted for the possibility that a predominance of the 12-kDa uncleaved form of p12^I^, observed in individuals with p12^I^ mutant (G29 → S), would cause an increase in HTLV-1 proviral load [11].

It was also observed that the wild type p12^I^G29, but not the mutate p12^I^S29, induces a decrease in tax-mediated transactivation. A recent study showed that different* ORF-I* mutations are associated with distinct patterns of p12 and p8 expression. While some mutations are associated with a balanced expression of p8 and p12, some* ORF-I* mutations result in a predominance of p8 or p12. As expected, the mutation G29S was associated with a predominance of p12 expression. However, in the same study, G29S virus was not infectious in macaques and was poorly infectious in monocytes and HTLV-1 infected T CD4+ cells by this mutant virus were partially susceptible to CTL killing. The same study showed that the proviral load was higher in individuals with the balanced expression of those proteins. This study finally concluded that p8 and p12 are both required for HTLV-1 persistence and spread [47].

There are also different mutations that result in premature stop codons and consequently the production of truncated p12 proteins. This truncated p12 protein has been described in strains from different countries and is not dependent on clinical presentation, once it was described in asymptomatic, HAM/TSP, and ATL individuals. Interestingly, this truncated protein was also observed in STLV. Some authors even used this fact as an argument to the hypothesis that p12 protein is not essential for virus replication.

## 8. Perspectives of HTLV-1 ORF-I Encoded Protein Studies

Several studies did not evaluate isolated p12 and p8 protein functions. Consequently, the better understanding of the role and the importance of each protein on HTLV-1 infection becomes limited. Another issue is that a lot of knowledge in this field was obtained using animal model and cell culture. Animal model may not represent what really happens in natural human infections. This can be exemplified with the fact that G29S strain was not able to infect monkeys; however, it is circulating in human host [47]. Another example is the contradictory results obtained when using different animal models, such as non-human primates and rabbits [55]. As for* in vitro* study, the culture conditions, such as the phytohemagglutinin stimulation and the media supplementation with IL-2, may interfere in the obtained results [51]. As ORF-I encoded proteins are associated with alterations in IL-2R and IL-2 promoter, this is an important bias. In fact, Albrecht and colleagues showed that in the absence of IL-2 and mitogen stimuli, p12 deletion reduced virus transmission in PBMCs [46]. ORF-I encoded proteins expression seems to be important mainly in suboptimal antigen stimulation and at a low IL-2 concentration, when those proteins may confer a proliferative advantage to HTLV-1 infected cells.

The role of ORF-I proteins in immune evasion and T-cell proliferation highlight the importance of these proteins, ORF-I diversity, and the proviral load in HTLV-1 infected individuals. In addition, new studies should address asymptomatic and symptomatic patients (HAM/TSP and ATL). This information is extremely important since the proviral load is the main prognostic factor for the development of complications associated with HTLV-1 infection.

There are still few studies with controversial data regarding this theme. Many promising findings could help to achieve a better understanding regarding HTLV-1 pathogenesis and may help to identify a possible therapeutic target and vaccine. The disease progression in HTLV-1 infected individuals is a very complex issue. Viral, host, and environmental factors may contribute to clinical outcome. How these factors interact in each individual may determine the development of disease.

## References

[1] Poiesz B. J., Ruscetti F. W., Gazdar A. F., Bunn P. A., Minna J. D., Gallo R. C. (1980). Detection and isolation of type C retrovirus particles from fresh and cultured lymphocytes of a patient with cutaneous T-cell lymphoma. *Proceedings of the National Academy of Sciences of the United States of America*.

[2] Gessain A., Cassar O. (2012). Epidemiological aspects and world distribution of HTLV-1 infection. *Frontiers in Microbiology*.

[3] Cook L. B., Elemans M., Rowan A. G., Asquith B. (2013). HTLV-1: persistence and pathogenesis. *Virology*.

[4] Verdonck K., González E., Van Dooren S., Vandamme A.-M., Vanham G., Gotuzzo E. (2007). Human T-lymphotropic virus 1: recent knowledge about an ancient infection. *The Lancet Infectious Diseases*.

[5] Gessain A., Mahieux R. (2012). Tropical spastic paraparesis and HTLV-1 associated myelopathy: clinical, epidemiological, virological and therapeutic aspects. *Revue Neurologique*.

[6] Nagai M., Usuku K., Matsumoto W. (1998). Analysis of HTLV-I proviral load in 202 HAM/TSP patients and 243 asymptomatic HTLV-I carriers: high proviral load strongly predisposes to HAM/TSP. *Journal of Neurovirology*.

[7] Lezin A., Olindo S., Olière S. (2005). Human T lymphotropic virus type I (HTLV-I) proviral load in cerebrospinal fluid: a new criterion for the diagnosis of HTLV-I–associated myelopathy/tropical spastic paraparesis?. *Journal of Infectious Diseases*.

[8] Grassi M. F. R., Olavarria V. N., Kruschewsky R. D. A. (2011). Human T cell lymphotropic virus type 1 (HTLV-1) proviral load of HTLV-associated myelopathy/tropical spastic paraparesis (HAM/TSP) patients according to new diagnostic criteria of HAM/TSP. *Journal of Medical Virology*.

[9] Rosadas C., Cabral-Castro M. J., Vicente A. C. P., Peralta J. M., Puccioni-Sohler M. (2013). Validation of a quantitative real-time PCR assay for HTLV-1 proviral load in peripheral blood mononuclear cells. *Journal of Virological Methods*.

[10] Puccioni-Sohler M., Yamano Y., Rios M. (2007). Differentiation of HAM/TSP from patients with multiple sclerosis infected with HTLV-I. *Neurology*.

[11] Fukumoto R., Andresen V., Bialuk I. (2009). In vivo genetic mutations define predominant functions of the human T-cell leukemia/lymphoma virus p12I protein. *Blood*.

[12] Edwards D., Fenizia C., Gold H. (2011). Orf-I and Orf-II-encoded proteins in HTLV-1 infection and persistence. *Viruses*.

[13] Koralnik I. J., Gessain A., Klotman M. E., Monico A. L., Berneman Z. N., Franchini G. (1992). Protein isoforms encoded by the pX region of human T-cell leukemia/lymphotropic virus type I. *Proceedings of the National Academy of Sciences of the United States of America*.

[14] Puccioni-Sohler M., Rios M., Bianco C. (1999). An inverse correlation of HTLV-I viral load in CSF and intrathecal synthesis of HTLV-I antibodies in TSP/HAM. *Neurology*.

[15] Johnson J. M., Nicot C., Fullen J. (2001). Free major histocompatibility complex class I heavy chain is preferentially targeted for degradation by human T-cell leukemia/lymphotropic virus type 1 p12^I^ protein. *Journal of Virology*.

[16] Collins N. D., D'Souza C., Albrecht B. (1999). Proliferation response to interleukin-2 and Jak/Stat activation of T cells immortalized by human T-cell lymphotropic virus type 1 is independent of open reading frame I expression. *Journal of Virology*.

[17] Smyth M. J., Cretney E., Kelly J. M. (2005). Activation of NK cell cytotoxicity. *Molecular Immunology*.

[18] Chan C. J., Smyth M. J., Martinet L. (2014). Molecular mechanisms of natural killer cell activation in response to cellular stress. *Cell Death and Differentiation*.

[19] Banerjee P., Feuer G., Barker E. (2007). Human T-cell leukemia virus type 1 (HTLV-1) p12I down-modulates ICAM-1 and -2 and reduces adherence of natural killer cells, thereby protecting HTLV-1-infected primary CD4^+^ T cells from autologous natural killer cell-mediated cytotoxicity despite the reduction of major histocompatibility complex class I molecules on infected cells. *Journal of Virology*.

[20] Sawada M., Suzumura A., Yoshida M., Marunouchi T. (1990). Human T-cell leukemia virus type I trans activator induces class I major histocompatibility complex antigen expression in glial cells. *Journal of Virology*.

[21] Bai X. T., Nicot C. (2012). Overview on HTLV-1 p12, p8, p30, p13: accomplices in persistent infection and viral pathogenesis. *Frontiers in Microbiology*.

[22] Nicot C., Mulloy J. C., Ferrari M. G. (2001). HTLV-1 p12^I^ protein enhances STAT5 activation and decreases the interleukin-2 requirement for proliferation of primary human peripheral blood mononuclear cells. *Blood*.

[23] Van Prooyen N., Andresen V., Gold H., Bialuk I., Pise-Masison C., Franchini G. (2010). Hijacking the T-cell communication network by the human T-cell leukemia/lymphoma virus type 1 (HTLV-1) p12 and p8 proteins. *Molecular Aspects of Medicine*.

[24] Albrecht B., D'Souza C. D., Ding W., Tridandapani S., Coggeshall K. M., Lairmore M. D. (2002). Activation of nuclear factor of activated T cells by human T-lymphotropic virus type 1 accessory protein p12I. *Journal of Virology*.

[25] Ding W., Albrecht B., Kelley R. E. (2002). Human T-cell lymphotropic virus type 1 p12^I^ expression increases cytoplasmic calcium to enhance the activation of nuclear factor of activated T cells. *Journal of Virology*.

[26] Ding W., Albrecht B., Luo R. (2001). Endoplasmic reticulum and cis-Golgi localization of human T-lymphotropic virus type 1 p12^I^: association with calreticulin and calnexin. *Journal of Virology*.

[27] Albrecht B., Lairmore M. D. (2002). Critical role of human T-lymphotropic virus type 1 accessory proteins in viral replication and pathogenesis. *Microbiology and Molecular Biology Reviews*.

[28] Ding W., Kim S.-J., Nair A. M. (2003). Human T-cell lymphotropic virus type 1 p12I enhances interleukin-2 production during T-cell activation. *Journal of Virology*.

[29] Kim S.-J., Ding W., Albrecht B., Green P. L., Lairmore M. D. (2003). A conserved calcineurin-binding motif in human T lymphotropic virus type 1 p12I functions to modulate nuclear factor of activated T cell activation. *The Journal of Biological Chemistry*.

[30] Mulloy J. C., Crowley R. W., Fullen J., Leonard W. J., Franchini G. (1996). The human T-cell leukemia/lymphotropic virus type 1 p12 protein binds the interleukin-2 receptor *β* and *γ*c chains and affects their expression on the cell surface. *Journal of Virology*.

[31] Nicot C., Mulloy J. C., Ferrari M. G. (2001). HTLV-1 p12I protein enhances STAT5 activation and decreases the interleukin-2 requirement for proliferation of primary human peripheral blood mononuclear cells. *Blood*.

[32] Taylor J. M., Brown M., Nejmeddine M. (2009). Novel role for interleukin-2 receptor-jak signaling in retrovirus transmission. *Journal of Virology*.

[33] Lairmore M. D., Albrecht B., D'Souza C. (2000). In vitro and in vivo functional analysis of human T cell lymphotropic virus type 1 pX open reading frames I and II. *AIDS Research and Human Retroviruses*.

[34] Meekings K. N., Leipzig J., Bushman F. D., Taylor G. P., Bangham C. R. M. (2008). HTLV-1 integration into transcriptionally active genomic regions is associated with proviral expression and with HAM/TSP. *PLoS Pathogens*.

[35] Gillet N. A., Malani N., Melamed A. (2011). The host genomic environment of the provirus determines the abundance of HTLV-1-infected T-cell clones. *Blood*.

[36] Mortreux F., Kazanji M., Gabet A.-S., de Thoisy B., Wattel E. (2001). Two-step nature of human T-cell leukemia virus type 1 replication in experimentally infected squirrel monkeys (*Saimiri sciureus*). *Journal of Virology*.

[37] Majorovits E., Nejmeddine M., Tanaka Y., Taylor G. P., Fuller S. D., Bangham C. R. M. (2008). Human T-lymphotropic virus-1 visualized at the virological synapse by electron tomography. *PLoS ONE*.

[38] Pais-Correia A.-M., Sachse M., Guadagnini S. (2010). Biofilm-like extracellular viral assemblies mediate HTLV-1 cell-to-cell transmission at virological synapses. *Nature Medicine*.

[39] Dustin M. L., Bivona T. G., Philips M. R. (2004). Membranes as messengers in T cell adhesion signaling. *Nature Immunology*.

[40] Kim S.-J., Nair A. M., Fernandez S., Mathes L., Lairmore M. D. (2006). Enhancement of LFA-1-mediated T cell adhesion by human T lymphotropic virus type 1 p12I1. *Journal of Immunology*.

[41] Van Prooyen N., Gold H., Andresen V. (2010). Human T-cell leukemia virus type 1 p8 protein increases cellular conduits and virus transmission. *Proceedings of the National Academy of Sciences of the United States of America*.

[42] Lin H.-C., Hickey M., Hsu L., Medina D., Rabson A. B. (2005). Activation of human T cell leukemia virus type 1 LTR promoter and cellular promoter elements by T cell receptor signaling and HTLV-1 Tax expression. *Virology*.

[43] Collins N. D., Newbound G. C., Albrecht B., Beard J. L., Ratner L., Lairmore M. D. (1998). Selective ablation of human T-cell lymphotropic virus type 1 p12^I^ reduces viral infectivity in vivo. *Blood*.

[44] Derse D., Mikovits J., Ruscetti F. (1997). X-I and X-II open reading frames of HTLV-I are not required for virus replication or for immortalization of primary T-cells in vitro. *Virology*.

[45] Robek M. D., Wong F.-H., Ratner L. (1998). Human T-cell leukemia virus type 1 pX-I and pX-II open reading frames are dispensable for the immortalization of primary lymphocytes. *Journal of Virology*.

[46] Albrecht B., Collins N. D., Burniston M. T. (2000). Human T-lymphotropic virus type 1 open reading frame I p12(I) is required for efficient viral infectivity in primary lymphocytes. *Journal of Virology*.

[47] Pise-Masison C. A., de Castro-Amarante M. F., Enose-Akahata Y. (2014). Co-dependence of HTLV-1 p12 and p8 functions in virus persistence. *PLoS Pathogens*.

[48] Berneman Z. N., Gartenhaus R. B., Reitz M. S. (1992). Expression of alternatively spliced human T-lymphotropic virus type I pX mRNA in infected cell lines and in primary uncultured cells from patients with adult T-cell leukemia/lymphoma and healthy carriers. *Proceedings of the National Academy of Sciences of the United States of America*.

[49] Cereseto A., Berneman Z., Koralnik I., Vaughn J., Franchini G., Klotman M. E. (1997). Differential expression of alternatively spliced pX mRNAs in HTLV-I-infected cell lines. *Leukemia*.

[50] Dekaban G. A., Peters A. A., Mulloy J. C. (2000). The HTLV-I orfl protein is recognized by serum antibodies from naturally infected humans and experimentally infected rabbits. *Virology*.

[51] Trovato R., Mulloy J. C., Johnson J. M., Takemoto S., De Oliveira M. P., Franchini G. (1999). A lysine-to-arginine change found in natural alleles of the human T-cell lymphotropic/leukemia virus type 1 p12(I) protein greatly influences its stability. *Journal of Virology*.

[52] Martins M. L., Soares B. C., Ribas J. G. (2002). Frequency of p12K and p12R alleles of HTLV type 1 in HAM/TSP patients and in asymptomatic HTLV type 1 carriers. *AIDS Research and Human Retroviruses*.

[53] Iñiguez A. M., Gastaldello R., Gallego S., Otsuki K., Vicente A. C. P. (2006). HTLV-1 p12^I^ protein sequences from South America: truncated proteins and common genetic signatures. *AIDS Research and Human Retroviruses*.

[54] Iñiguez A. M., Otsuki K., Magalhães G. P., Silva E. A., Vicente A. C. P. (2005). Genetic markers on the HTLV-1 p12I protein sequences from Brazilian HAM/TSP patients and asymptomatic HTLV-1 carrier isolates. *AIDS Research and Human Retroviruses*.

[55] Valeri V. W., Hryniewicz A., Andresen V. (2010). Requirement of the human T-cell leukemia virus p12 and p30 products for infectivity of human dendritic cells and macaques but not rabbits. *Blood*.

